# Assessment of blood cobalt levels and safety of long-term hydroxocobalamin therapy in patients with cblC defect

**DOI:** 10.3389/fnut.2026.1843743

**Published:** 2026-07-30

**Authors:** Lili Hao, Yi Ding, Yuxin Deng, Ting Chen, Xia Zhan, Feng Xu, Haijuan Zhi, Kaichuang Zhang, Yi Yang, Ruifang Wang, Yuning Sun, Lianshu Han

**Affiliations:** Department of Pediatric Endocrinology and Genetic Metabolism, Xinhua Hospital, Shanghai Institute for Pediatric Research, Shanghai Jiao Tong University School of Medicine, Shanghai, China

**Keywords:** adverse drug reactions, cobalt, hydroxocobalamin, methylmalonic acidemia, *MMACHC*

## Abstract

**Background:**

Hydroxocobalamin (OHCbl), the most effective form of vitamin B12 containing cobalt (Co) as its core component, is the mainstay therapy for cobalamin C (cblC) defect, yet long-term Co exposure and safety signals remain incompletely characterized.

**Methods:**

We retrospectively analyzed 379 cblC patients receiving OHCbl therapy. Among them, 270 had prognostic data and 99 had laboratory and Co measurements. Adverse drug reactions (ADRs) were evaluated using the Naranjo scale. Regression models identified determinants of Co levels and associations of OHCbl dosage, treatment duration, and Co concentration with routine laboratory parameters, potential ADRs and prognosis.

**Results:**

Median treatment duration was 76.70 months and median OHCbl dosage was 1.07 mg/kg/week; Median blood Co concentration was 22.53 μg/L. Although 53.54% of patients having Co >20.00 μg/L, no severe Co toxicity was observed. OHCbl dosage was the only independent predictor of Co (β = 0.70, *p* < 0.001). In adjusted analyses of 29 routine laboratory indicators, no association with OHCbl dosage, treatment duration, or blood Co remained significant after Benjamini–Hochberg false discovery rate correction (all *q* > 0.05). ADRs occurred in 82.6% of patients, most commonly chromaturia (80.39%), psychomotor agitation (8.84%), and localized rash (5.52%), and were predominantly mild and self-limited. Higher OHCbl dosage was associated with increased odds of any potential ADR (OR = 3.61) and chromaturia (OR = 3.00); longer treatment duration was independently associated with better overall prognosis (OR = 1.02), and no associations with injury to specific organ systems were detected.

**Conclusion:**

OHCbl remains well tolerated even with prolonged, higher-dosage treatment, supporting its continued long-term use with routine monitoring.

## Introduction

Combined methylmalonic acidemia and homocystinuria, cblC type (OMIM #277400, cblC defect), represents the most prevalent inherited disorder of intracellular cobalamin (vitamin B12) metabolism (1). Its worldwide incidence is about 1 in 100,000 live births but higher in China (1 in 39,000–1 in 3,500) (2, 3). The cblC defect is primarily attributed to the biallelic pathogenic variants in the *MMACHC* gene, which impair the intracellular conversion of dietary cobalamin into methylcobalamin and adenosylcobalamin, the cofactors for methionine synthase and methylmalonyl-CoA mutase (4, 5). This metabolic block results in the accumulation of homocysteine (HCY), methylmalonic acid (MMA), and methylcitrate (MCA), alongside normal or low methionine (MET) levels.

The cblC defect exhibits a broad clinical spectrum, with onset ranging from prenatal to adult life and multiorgan involvement affecting the central nervous, ocular, renal, hematologic, and cardiac systems (6). The *MMACHC* genotypes strongly influence disease onset and severity. Truncating or frameshift variants, such as c.609G>A and c.331C>T, are usually associated with early-onset disease within the first year of life and more severe clinical manifestations, whereas missense variants, such as c.80A>G and c.482G>A, are typically linked to late-onset disease after 1 year of age (7–10). Diagnosis, assessment of disease severity, and prognostic evaluation rely on metabolic indicators such as blood propionylcarnitine (C3), the C3/acetylcarnitine (C2) ratio, MET, HCY, urinary MMA (uMMA) and MCA (uMCA) (6). Tandem mass spectrometry-based newborn screening (NBS) enables early detection and timely treatment of cblC disease, thereby improving prognosis.

Intramuscular hydroxocobalamin (OHCbl) is the cornerstone of cblC treatment, often combined with betaine, L-carnitine, and folic acid (1, 11). Despite a recommended starting dose of 1 mg daily, the optimal long-term maintenance dose remains undefined (11). Previous studies suggest that higher OHCbl dosages (0.3–0.8 mg/kg/day) may improve neurocognitive outcomes (12, 13), but the long-term systemic effects and safety of prolonged high-dose therapy are unclear. Adverse events, including oxalate nephropathy (14), methemoglobinemia (15), myocardial injury (16), and hypertension (17), have been observed in patients or animal models following acute high-dose OHCbl administration for cyanide poisoning. Nevertheless, causal relationships are unconfirmed.

Cobalt (Co), which constitutes 4.5% of the cobalamin molecule, is essential for its pharmacological activity. Normal blood Co levels range from 0.1 to 1.2 μg/L (18, 19), whereas chronic elevations above 20 μg/L may be associated with systemic toxicity (20). Excessive Co exposure, either from oral supplementation or metal-on-metal hip implants, has been linked to skin, cardiovascular, endocrine, hematologic and neurological complications (20, 21). However, whether long-term OHCbl therapy leads to Co accumulation in patients with cblC deficiency and whether such accumulation has clinical consequences remain largely unknown.

This retrospective study aimed to evaluate the safety of prolonged OHCbl treatment, characterize the distribution and determinants of blood cobalt levels, and examine the associations of OHCbl therapy and blood Co levels with routine laboratory parameters, potential adverse drug reactions (ADRs) and prognosis. These findings may provide practical insights for optimizing the management of cblC deficiency.

## Methods

### Patients and data collection

From June 2024 to June 2025, a total of 379 patients (209 males and 170 females) with a confirmed cblC defect under long-term OHCbl therapy were included. According to data completeness and analytical purpose, patients were categorized into three overlapping subsets (Supplementary Figure S1 and Supplementary Table S1). The ADR Cohort (*n* = 379) included all patients with demographic, treatment, and ADR information and was used to assess the incidence and spectrum of ADRs during OHCbl therapy. The Clinical Outcome Cohort (*n* = 270) included patients with additional available data on clinical and prognostic information and was used to analyze the associations of OHCbl treatment with prognosis and ADRs. The Cobalt Cohort (*n* = 99) comprised patients who agreed to additional blood Co testing and had corresponding laboratory and imaging data available, enabling the assessment of Co determinants and relationships between OHCbl treatment, Co levels and laboratory parameters. No investigator-defined selection criteria were applied to either subset. Written informed consent was obtained from the parents of study participants. The study was conducted in accordance with the Helsinki Declaration and approved by the Ethics Committee of Xinhua Hospital Affiliated to Shanghai Jiao Tong University School of Medicine (Approval No. XHEC-D-2026-040).

### Biochemical tests

Metabolic markers (MET, C3, C2, uMMA and uMCA) were examined by standard tandem mass spectrometry or gas chromatography-mass spectrometry methods in the clinical laboratory (22). Plasma HCY levels were determined using a fluorescence polarization immunoassay. The Co concentrations in human whole blood were measured using inductively coupled plasma mass spectrometry (Agilent, USA) following standard protocols (23).

### Genotyping and grouping of *MMACHC* variants

Genomic DNA from peripheral blood was analyzed by Sanger sequencing or next-generation sequencing. Variants were annotated with reference to the *MMACHC* transcript (NM_015506) from the NCBI GenBank database. Pathogenicity classifications were based on the guidelines of the American College of Medical Genetics and Genomics (ACMG) (24). According to the genotype-phenotype and genotype-biochemical correlations (8–10), patients were divided into four groups (A-D): A, truncating/indel variants; B, carrying c.80A>G (without c.482G>A); C, carrying c.482G>A; and D, other missense variants including c.394C>T.

### Treatment

Intramuscular OHCbl therapy was initiated with a five-day course of intramuscular OHCbl at a daily dose of 5–10 mg. After stabilization, both the dosage and administration frequency were adjusted individually according to patient age, weight, and clinical condition, with long-term OHCbl therapy ranging from 1 to 20 mg per administration at intervals of 1–20 days. In addition, oral medications such as betaine (100–300 mg/kg/day), L-carnitine (50–100 mg/kg/day), and/or folic acid (5–15 mg/day) were prescribed post-diagnosis as part of routine management.

### Investigation and evaluation of potential ADRs

ADRs were assessed using a structured questionnaire and supplemented by telephone follow-up for clarification when multiple events were reported. Based on previously reported safety data (17), six preselected event types were evaluated: urine discoloration, neuropsychiatric symptoms, dermatologic reactions, gastrointestinal symptoms, hypertension, and severe allergic events. Reported events were analyzed as potential ADRs using the Naranjo scale, which provided a structured causality assessment rather than definitive proof of drug causality. For each event type, Naranjo scores were calculated among patients reporting that event, and the median score was used to assign an overall causality category: definite (≥9), probable (5–8), possible (1–4), or doubtful ( ≤ 0).

### Follow-up and outcome evaluations

Patients were regularly followed in the clinic. Follow-up visits were initially scheduled monthly and were later extended to every 1–3 months after clinical stabilization. Assessments included treatment adherence, growth and developmental progress, metabolic parameters, routine laboratory tests, imaging studies, and cognitive or neurological evaluations. Standardized developmental or cognitive assessments, including the Gesell Developmental Schedules and age-appropriate Wechsler Intelligence Scales, were performed when available. Outcome assessment was not formally blinded to OHCbl exposure or blood Co levels. Prognosis was first evaluated by treating clinicians during routine follow-up. For this retrospective study, investigators extracted prognostic information from medical records and categorized outcomes according to predefined criteria. A good prognosis was defined as the absence of major organ dysfunction and normal developmental status at the last visit. A poor prognosis was defined as clinically significant dysfunction in one or more organ systems, including cardiovascular, renal, hepatic, hematologic, endocrine, ocular, or nervous system involvement. For patients without complete examinations across all organ systems, prognosis was classified based on the available medical records, treating clinicians' assessments, laboratory results, imaging findings, developmental or neurological assessments, and clinical functional status. The criteria used to define organ-specific involvement and overall prognosis are detailed in the Supplementary Methods.

### Statistical analysis

Most statistical analyses were performed using SPSS version 26.0 (SPSS Inc., Chicago, IL) and GraphPad Prism Software 8.0 (Dotmatics, Boston, MA). Continuous variables were presented as mean ± standard deviation (SD) or median (interquartile range, IQR), and categorical variables are presented as counts and percentages. Group comparisons were performed using Student's *t*-test or the Mann–Whitney U test for continuous variables and the chi-square or Fisher's exact test for categorical variables, as appropriate.

Missing data were not imputed. Descriptive summaries and group comparisons were based on the available data for each variable. Conventional regression models were performed using complete-case analysis, in which participants with missing values for any required outcome, exposure, predictor, or covariate were excluded. For LASSO analyses, missing categorical predictors were encoded as an additional “unknown” category, while observations with missing outcomes or missing continuous covariates were excluded. The sample size for each analysis is reported in the corresponding tables or figure legends.

Multiple linear regression was used to identify determinants of blood Co concentration and to assess the associations of treatment-related exposures (OHCbl dosage, treatment duration, and blood Co) with laboratory indicators. For blood Co concentration, a multivariable linear regression model was fitted with OHCbl dosage and treatment duration as the main exposures, with adjustment for age, sex, disease onset status, *MMACHC* variant type, and baseline liver and kidney impairment. For laboratory indicators, 29 indicators listed in Supplementary Table S2 were analyzed using prespecified multivariable regression models including OHCbl dosage, treatment duration, and blood Co concentration as treatment-related exposures. These models were adjusted for sex, age, *MMACHC* variant type, disease onset, and concurrent metabolic indicators (MMA, HCY). Multiple testing across laboratory indicators was controlled using the Benjamini–Hochberg false discovery rate (BH-FDR) procedure applied to exposure–indicator tests, with 29 tests per exposure and 87 tests in total. A *q* value < 0.05 was considered significant.

For ADRs and prognosis, candidate predictors included OHCbl dosage, treatment duration, blood Co concentration, sex, age, *MMACHC* variant type, disease onset, NBS status, and concurrent metabolic indicators including C3, C3/C2, MET, MMA, MCA, and HCY. These predictors were first screened using least absolute shrinkage and selection operator (LASSO) regression with the glmnet package in R. The optimal penalty parameter, λ, was selected by 10-fold cross-validation. Variables retained by LASSO were subsequently included in multivariable logistic regression models.

## Results

### Cohort and OHCbl exposure overview

A total of 379 cblC patients (209 males and 170 females) were included in the ADR analysis of OHCbl (ADR cohort). Among them, clinical and prognostic data at the final follow-up visit were available for 269 participants (Clinical Outcome Cohort). Concurrent blood Co and core laboratory data were available for 99 of these subjects (Cobalt Cohort). Across the ADR cohort, the median treatment duration was 76.7 months (IQR: 31.38–114.30) and the median OHCbl dosage was 1.07 mg/kg/week (IQR: 0.44–1.90). Baseline characteristics of the three overlapping cohorts were compared in Supplementary Table S1. Overall, the cohorts showed no major baseline imbalance, although treatment duration, ADR-related variables, and the proportion of neurological abnormalities among patients with poor prognosis differed among cohorts.

### Blood Co levels and associated factors

Co is the core component of OHCbl, and excessive accumulation may lead to multi-system toxicities (20). Thus, blood Co levels were firstly examined in patients from the Cobalt Cohort. The median Co concentration was 22.53 μg/L (IQR: 8.03–38.72). Patients were categorized into a low-Co group ( ≤ 20 μg/L) and a high-Co group (>20 μg/L). Before adjustment, patients in the high-Co group received higher OHCbl dosages, had a greater proportion of *MMACHC* A-type variants, were more frequently classified as early-onset cases and exhibited a worse prognosis (all *p* < 0.05, Table 1, placed at the end of the text file).

**Table 1:** Clinical features of cblC patients according to Co levels before and after PSM.

Characteristics	Before PSM				After PSM			
	Overall	Low-Co	High-Co	*p* value	Overall	Low-Co	High-Co	*p* value
*n*.	99	46	53		34	17	17	
Male sex, *n* (%)	56 (56.57)	27 (58.70)	29 (54.72)	0.69	18 (52.94)	9 (52.94)	9 (52.94)	>0.9999
Female sex, *n* (%)	43 (43.43)	19 (41.30)	24 (45.28)		16 (47.06)	8 (47.06)	8 (47.06)	
Age (months), median (IQR)	84.00 (29.97–139.20)	96.49 (26.23–172.20)	78.03 (40.93–123.60)	0.3196	101.60 (66.76–144.60)	116.50 (65.98–164.20)	92.90 (56.00–138.10)	0.9632
*MMACHC* variants^*^								
A, *n* (%)	43 (45.26)	11 (23.91)^a^	33 (66.00)^b^	< 0.0001	19 (57.58)	9 (52.95)	10 (62.50)	0.666
B, *n* (%)	17 (17.89)	5 (10.87)^a^	12 (24.00)^a^		9 (27.27)	4 (23.53)	5 (31.25)	
C, *n* (%)	26 (27.37)	24 (52.18)^a^	2 (40.00)^b^		2 (6.06)	2 (11.76)	0 (0.00)	
D, *n* (%)	9 (9.48)	6 (13.04)^a^	3 (6.00)^a^		3 (9.09)	2 (11.76)	1 (6.25)	
Onset of disease								
Disease onset, *n* (%)	69 (70.41)	25 (54.35)	45 (84.91)	0.001	27 (79.41)	11 (64.71)	16 (94.12)	0.0854
Late onset, *n* (%)	22 (33.33)	13 (52.00)	9 (21.95)	0.012	7 (19.17)	4 (40.00)	3 (21.43)	0.3926
Onset age (months), median (IQR)	6.00 (1.37–24.33)	24.33 (2.37–129.90)	3.47 (0.97–8.25)	0.0162	3.77 (0.96–14.47)	6.00 (1.33–41.13)	3.24 (0.72–8.42)	0.4375
Diagnosis								
NBS, *n* (%)	48 (47.96)	25 (54.35)	23 (43.40)	0.277	14 (41.18)	8 (47.06)	6 (35.29)	0.7283
Treatment								
Duration of treatment (months), median (IQR)	42.77 (11.40–90.00)	28.62 (9.57–75.81)	49.70 (17.48–107.5)	0.0771	73.19 (25.50–112.20)	70.60 (8.67–99.22)	86.03 (40.39–123.80)	0.2794
Dosage (mg/kg/week), median (IQR)	1.22 (0.62–1.90)	0.57 (0.26–0.96)	1.69 (1.25–2.21)	< 0.0001	1.22 (0.79–1.68)	1.18 (0.83–1.74)	1.25 (0.78–1.72)	0.491
Co concentration (μg/l), 15.6-7.2,-1.3690ptmedian (IQR)	21.74 (7.66–38.40)	6.17 (1.14–12.87)	37.55 (25.86–67.51)	< 0.0001	21.25 (13.62–31.93)	13.76 (10.06–17.93)	31.06 (24.10–41.67)	< 0.0001
Outcomes								
Good prognosis, *n* (%)	25 (28.09)	17 (38.64)	8 (17.78)	0.0352	10 (38.24)	7 (41.18)	3 (21.43)	0.2802
Disorders of the								
Cardiovascular system, *n* (%)	5 (6.33)	5 (11.90)	0 (0.00)	0.0571	1 (3.13)	1 (5.88)	0 (0.00)	>0.9999
Hematologic system, *n* (%)	3 (3.23)	2 (4.44)	1 (2.08)	0.6088	0 (0.00)	0 (0.00)	0 (0.00)	>0.9999
Liver, *n* (%)	5 (5.38)	3 (6.52)	2 (4.26)	0.6771	0 (0.00)	0 (0.00)	0 (0.00)	>0.9999
Kidneys, *n* (%)	5 (5.88)	3 (7.14)	2 (4.65)	0.676	3 (9.09)	1 (6.25)	2 (11.76)	>0.9999
Endocrine system, *n* (%)	4 (5.88)	3 (9.09)	1 (2.86)	0.349	0 (0.00)	0 (0.00)	0 (0.00)	>0.9999
Nervous system, *n* (%)	54 (78.26)	22 (73.33)	32 (82.05)	0.3973	19 (73.08)	9 (69.23)	10 (76.92)	>0.9999
Adverse drug reactions								
Urine red staining, *n* (%)	57 (67.86)	24 (58.54)	33 (76.74)	0.074	23 (76.67)	12 (80.00)	11 (73.33)	>0.9999
Psychomotor agitation, *n* (%)	3 (3.61)	2 (4.88)	1 (2.38)	0.616	0 (0.00)	0 (0.00)	0 (0.00)	>0.9999
Localized rash, *n* (%)	3 (3.61)	0 (0.00)	3 (7.14)	0.241	1 (3.45)	0 (0.00)	1 (6.67)	>0.9999
None, *n* (%)	25 (30.12)	16 (39.02)	9 (21.43)	0.081	7 (23.33)	3 (20.00)	4 (26.67)	>0.9999

Co, Cobalt; PSM, Propensity score matching; n, number; IQR, interquartile range; NBS, newborn screening.

^*^Groups sharing a common superscript letter (a, b) do not differ significantly (*p* > 0.05), the letter-based comparisons (a, b) are only valid within the same subset row.

**Figure 1:**
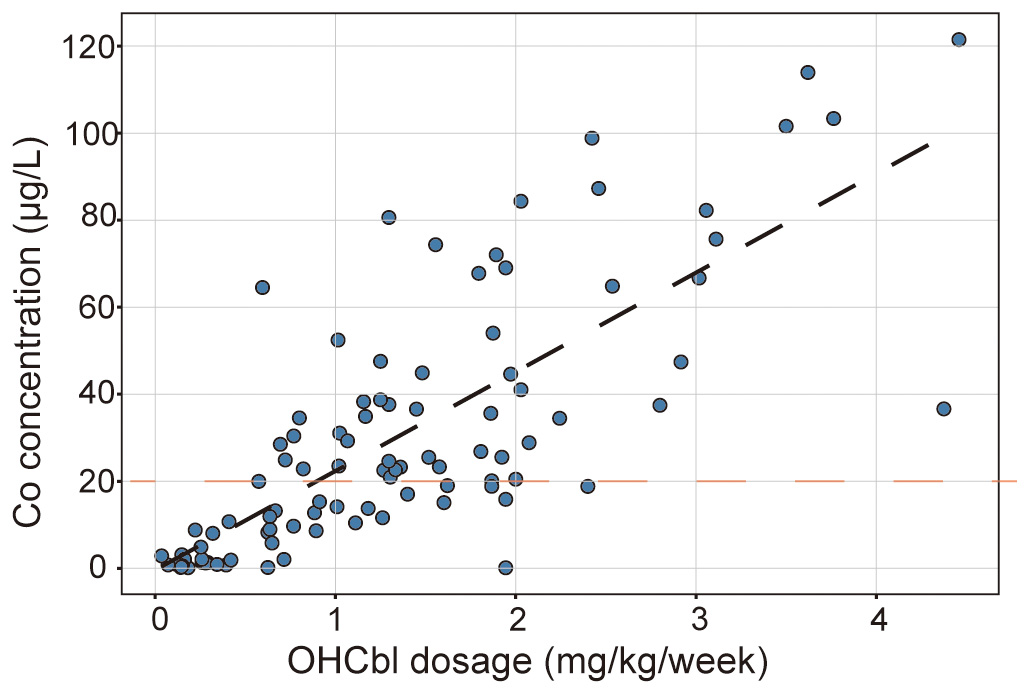
Association between OHCbl dosage and blood Co concentration. The Scatter plot shows the correlation between OHCbl dosage and blood Co concentration (*n* = 98). The black dashed line represents the fitted regression trend.

A multivariable linear regression model was then fitted to identify factors independently associated with blood Co concentration. After adjustment for prespecified covariates (see Methods), only OHCbl dosage remained independently associated with Co levels (β = 0.696, *p* < 0.001; Figure 1 and Supplementary Table S2).

### Associations between Co levels and laboratory parameters

Although 53 patients had blood Co concentrations >20 μg/L, no clinically apparent signs of severe Co toxicity were observed. We therefore examined associations between Co levels and routine laboratory parameters to identify potential monitorable safety signals. Compared with the low-Co group, the high-Co group showed lower red cell counts, mean platelet volume and uric acid (UA) levels, but higher lactate dehydrogenase levels and blood urea nitrogen-to-creatinine ratios (Supplementary Table S3). Given that Co levels were strongly associated with OHCbl dosage, propensity score matching (PSM, 1:1) was applied to balance OHCbl dosage between the two groups, yielding 17 matched pairs. After matching, most baseline characteristics and laboratory parameters were comparable between groups, although UA levels remained lower in the high-Co group (Table 1 and Supplementary Table S3). However, the proportion of patients with subnormal UA levels remained low (14.89% before and 12.50% after matching) and did not differ significantly between groups, with no significant linear correlation between Co and UA levels (*p* < 0.05). Because only 17 matched pairs were available after PSM, the matched analysis was underpowered and should be interpreted as exploratory. Further validation of the relationship between Co and UA levels requires larger longitudinal cohorts.

### Impact of OHCbl treatment on laboratory profiles

As supportive safety monitoring, we explored whether OHCbl exposure metrics, including dosage and treatment duration, and blood Co concentration were independently associated with routine laboratory profiles after adjustment for sex, age, *MMACHC* variant type, onset status and concurrent metabolic indicators, including uMMA and HCY (Supplementary Table S4). Among the 29 laboratory indicators analyzed, three exposure-indicator associations reached nominal significance in multivariable models (*p* < 0.05). Higher Co concentration was associated with higher blood urea nitrogen (BUN), whereas longer treatment duration was associated with higher gamma-globulin (GAMMA) levels and lower white blood cell (WBC) counts (Table 2). However, after BH-FDR correction for multiple testing across 87 exposure-indicator tests, none of these associations remained statistically significant (all *q* > 0.05).

**Table 2:** Significant associations between OHCbl exposure metrics and laboratory indicators.

Laboratory indicator	Exposure metric	*n*	B^*^	SE	*t*	*p*-value^#^	*q*-value^#^
BUN (mg/dL)	Co concentration (μg/L)	73	0.46	0.01	2.97	0.004	0.35
GAMMA (g/L)	Treatment duration (months)	73	0.28	0.009	2.46	0.017	0.74
WBC ( × 10^9^/L)	Treatment duration (months)	75	−0.30	0.005	−2.11	0.039	0.97

^*^β represents the change in the laboratory indicators per 1-unit increase in the exposure metric. ^#^*p*-values are raw model-derived *p*-values; *q*-values were BH-FDR-adjusted values calculated for exposure–outcome tests across 29 laboratory outcomes (29 tests per exposure; 87 tests in total for dosage, Co, and treatment duration).

### Association of OHCbl treatment with clinical outcomes

**Figure 2:**
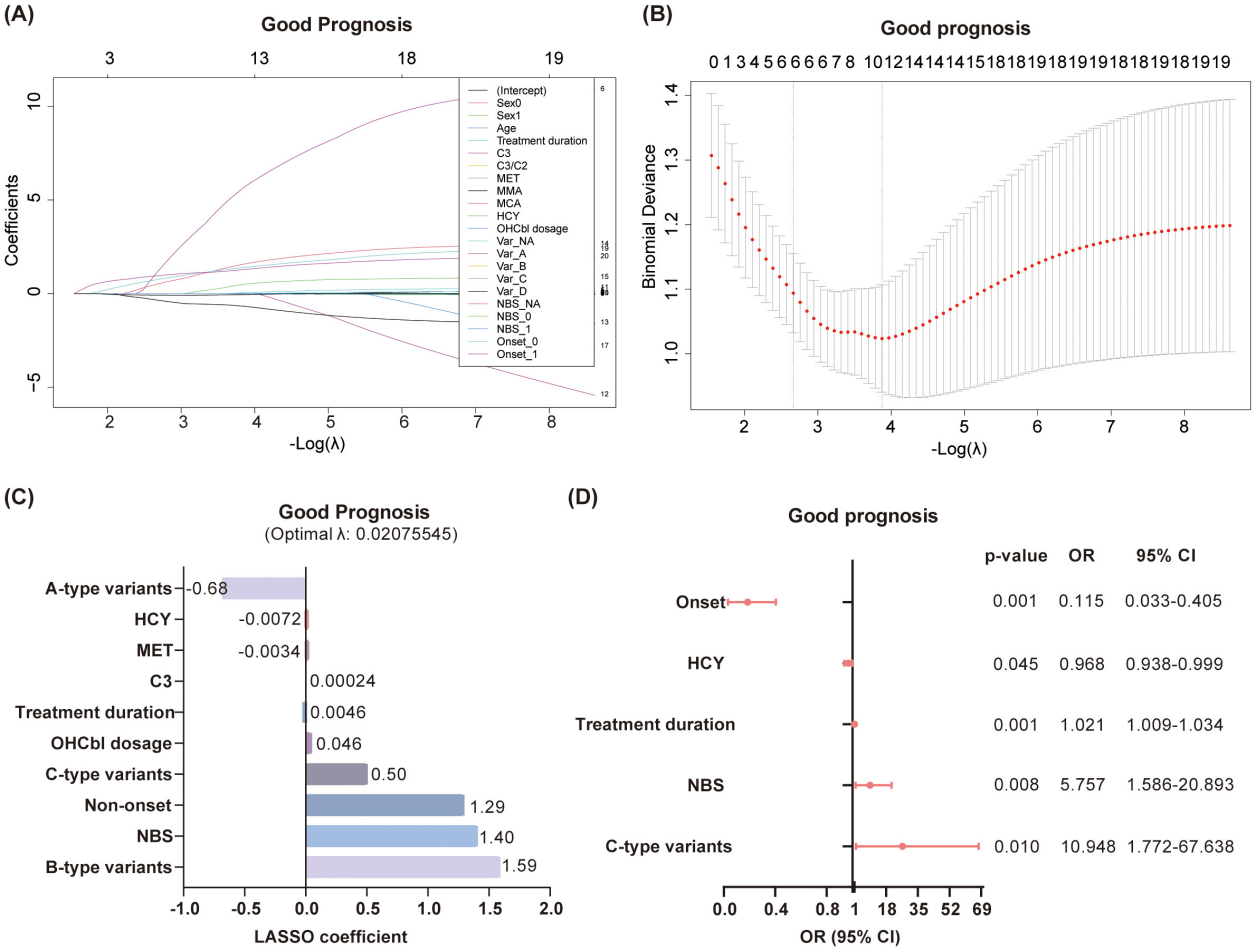
Predictors associated with good prognosis. **(A)** Coefficient path plot shows trajectories of predictor coefficients across –log (λ) values, shrinking toward zero with increasing λ. **(B)** Cross-validation plots display binomial deviance vs. –log (λ), with red dots indicating mean deviance, gray bands representing standard errors, and dashed lines marking optimal λ values. **(C)** Bar chart showing variables selected through LASSO regression with their corresponding coefficients and the optimal λ value identified by cross-validation. Candidate predictors included OHCbl dosage, treatment duration, sex, age, *MMACHC* variant type, disease onset, NBS status, and metabolic indicators including C3, C3/C2, MET, MMA, MCA, and HCY. *n* = 117. **(D)** Forest plot showing predictor effects on good prognosis analyzed by binary logistic regression. The binary logistic regression was mutually adjusted for the LASSO-selected variables shown in panel **C**. *n* = 136. Data are presented as median with range.

Because the laboratory analyses suggested potential associations between OHCbl-related exposure metrics and several organ function-related indicators, we further explored their associations with clinical prognosis. LASSO regression identified treatment duration, OHCbl dosage, *MMACHC* variant types, NBS status, disease onset and metabolic indicators (C3, MET, and HCY) as candidate predictors of favorable outcomes (Figures 2A-C). Subsequent multivariate logistic regression showed that longer OHCbl treatment duration [odds ratio (OR) =1.021], the *MMACHC* C-type variant (c.482G>A) and NBS were independently associated with better prognosis, whereas disease onset status was negatively associated with better prognosis (Figure 2D). No significant associations were observed between OHCbl therapy and injury to specific organs (heart, liver, kidney, brain, blood and thyroid). However, these organ-specific analyses were limited by low event numbers and should be interpreted cautiously.

### Potential ADRs of OHCbl

To further investigate the safety profile of long-term OHCbl therapy, we summarized potential ADRs and evaluated their patterns and predictors. Among the 379 enrolled patients, 315 (82.60%) exhibited at least one potential ADR. As shown in Table 3, the three most common events were chromaturia (*n* = 291, 80.39%), psychomotor agitation (*n* = 32, 8.84%) and localized rash (*n* = 20, 5.52%). According to the Naranjo Assessment Scale (25), chromaturia was classified as “certain” (median score, 9.0). Localized rash (median score, 6.0), abdominal pain or diarrhea (median score, 5.0), psychomotor agitation (median score, 5.0) and severe allergic reactions (median score, 5.0) were classified as “probable”, whereas elevated blood pressure was classified as “possible” (median score, 1.0). Collectively, these findings suggest that the recorded events may represent potential ADRs related to OHCbl, although causality should be interpreted cautiously given the retrospective design. Analyses of uncommon potential ADRs, including elevated blood pressure, abdominal pain and diarrhea, and severe allergic reactions, were underpowered because each subgroup included fewer than 10 patients; therefore, subgroup comparisons and predictor analyses for these events were considered exploratory.

**Table 3:** Potential ADRs of OHCbl and corresponding Naranjo assessment scale results.

Type of potential ADR	No. (%)	Naranjo score, median (IQR)	Naranjo score categories	OHCbl dosage (mg/kg/week), median (IQR)^*^
Chromaturia	291 (80.39)	9.00 (8.00–9.00)	Certain	1.15 (0.61–1.94)^a^
Psychomotor agitation	32 (8.84)	5.00 (4.00–6.00)	Probable	1.12 (0.52–2.13)^a^
Localized rash	20 (5.52)	6.00 (1.75–6.25)	Probable	0.93 (0.38–2.03)^a, b^
Elevated blood pressure	5 (1.38)	1.00 (1.00–5.50)	Possible	0.62 (0.16–0.96)^a, b^
Abdominal pain and diarrhea	3 (0.83)	6.00 (5.00–7.00)	Probable	0.93 (0.70–1.00)^a, b^
Severe allergic reaction	2 (0.55)	5.00, 5.00	Probable	0.94 (0.89–1.00)^a, b^
None	63 (17.40)	0	NA	0.46 (0.20–0.96)^b^

ADRs, adverse drug reactions; No, number; IQR, interquartile range; OHCbl, hydroxocobalamin. ^*^Groups sharing a common superscript letter (a, b) do not differ significantly (*p* > 0.05), the letter-based comparisons (a, b) are only valid within the same subset column.

**Figure 3:**
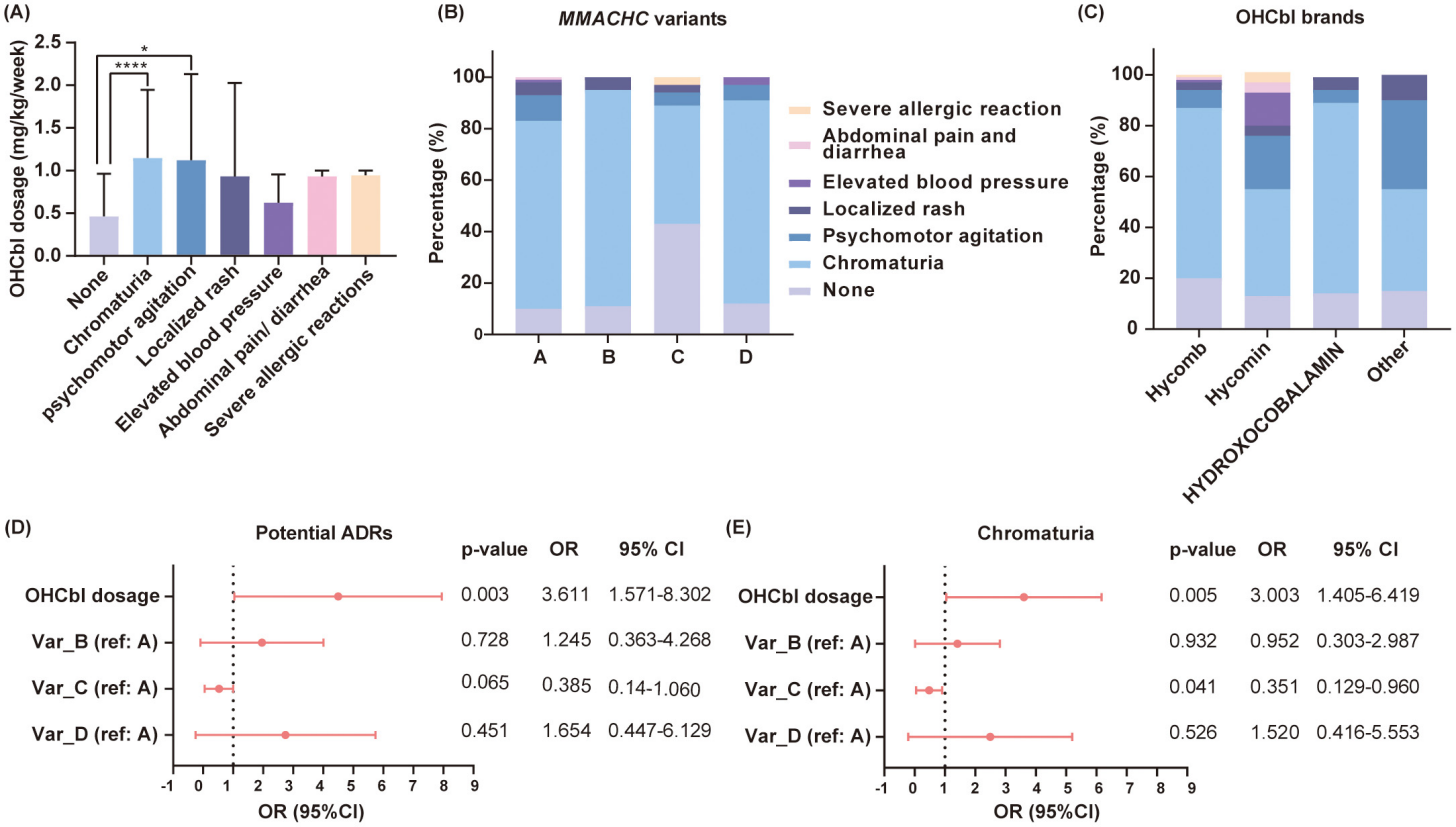
OHCbl dosage, brands, and *MMACHC* variants in relation to potential ADRs. **(A)** Bar chart showing OHCbl dosage (mg/kg/week) in patients with or without potential ADRs. Data are presented as median with IQR, with significance levels indicated (*p < 0.05; ****p < 0.0001). **(B)** Stacked bar chart showing ADR patterns stratified by *MMACHC* variants. **(C)** Stacked bar chart illustrating the proportion of ADRs associated with different OHCbl brands. **(D, E)** Forest plots showing predictor effects on potentially ADRs **(D)** and chromaturia **(E)** analyzed by the binary logistic regression. Candidate predictors included OHCbl dosage, age, MMACHC variant type and OHCbl brands that selected by LASSO regression. *n* = 215. Data are presented as median with range.

The OHCbl dosage, distribution of *MMACHC* variants and OHCbl brands were compared respectively between patients with or without different potential ADRs. Patients experiencing chromaturia or psychomotor agitation received significantly higher dosages than those without potential ADRs (Figure 3 and Table 3). The MMACHC c.482G>A variant appeared more frequent in the “None” group (42.11% vs. 10.53–36.84% for other variants) but less frequent in the “Chromaturia” group (9.71% vs. 15.43–56.57% for other variants; Figure 3 and Supplementary Table S5). In comparisons by OHCbl brand, 52.7% of patients with chromaturia received “HYDROXOCOBALAMIN”, compared with 4.8% receiving “Hycomin” or other OHCbl brands. Conversely, 60.0% of patients with elevated blood pressure received “Hycomin”, compared with 0%−40% for other brands (Figure 3 and Supplementary Table S5).

Furthermore, the LASSO regression and multivariate logistic regression modeling were applied to identify predictors of potential ADRs. Higher OHCbl dosage was linked to an increased risk of general potential ADRs (OR = 3.611) and chromaturia (OR = 3.003), whereas the *MMACHC* c.482A>G variant was associated with a lower risk of chromaturia (OR = 0.351, Figure 3 and Supplementary Figures S2, S3).

## Discussion

OHCbl, the most effective form of vitamin B12, exerts a pivotal therapeutic effect in cblC disease by increasing intracellular cobalamin concentrations and enhancing residual enzyme activity (11). High-dose OHCbl therapy has been reported to improve biochemical parameters and neurodevelopmental outcomes (13, 26), but most prior studies were limited by small sample sizes, leaving the efficacy and safety of long-term high-dose regimens uncertain. This large retrospective real-world cohort provides additional data on the long-term safety profile and clinical outcomes of prolonged OHCbl treatment in patients with cblC defect.

As OHCbl contains Co as its central element, chronic exposure through sustained treatment may lead to elevated circulating Co levels, raising concerns regarding its health implications. In the Cobalt Cohort, the median blood Co concentration was 22.53 μg/L, with approximately 50% of patients exceeding 20 μg/L, a threshold previously reported to potentially induce systemic toxicity (20). However, no severe clinical signs of Co toxicity were observed. Multivariable linear regression identified OHCbl dosage as the only independent predictor of Co levels, indicating that Co accumulation was primarily dose-related. Before OHCbl dosage balancing, patients with different Co levels differed in *MMACHC* variant types, disease onset status and overall prognosis. These differences were largely attenuated after PSM, suggesting that they were more likely related to dose variation rather than to a direct effect of Co itself. This interpretation is also consistent with our previous finding that variant type and disease status are independent determinants of OHCbl dose levels (8).

Several laboratory differences were observed in relation to Co levels or OHCbl exposure, but their clinical relevance appeared limited. The high-Co group showed significant lower UA levels after balanced the OHCbl dosage between the two group by PSM, and elevated Co levels were nominally associated with increased BUN. In addition, longer treatment duration was associated with higher GAMMA and lower WBC count. However, none of the 87 exposure-indicator tests remained significant after BH-FDR correction. BUN can be influenced by hydration status, dietary protein intake, and catabolic state and does not necessarily indicate intrinsic renal injury (27, 28). Likewise, modest shifts in GAMMA and WBC may reflect intercurrent inflammation/infection, immune status, or overall disease control over prolonged follow-up rather than organ toxicity attributable to OHCbl or Co accumulation (29). What's more, these parameters exhibited low abnormality rates (< 10%) and generally small deviations. Overall, we did not identify robust laboratory toxicity signals after multiplicity correction. Although blood Co testing was voluntary and based on data availability, no investigator-defined selection criteria were applied, and Supplementary Table S1 did not show broad baseline imbalance among the cohorts. Nevertheless, analyses involving Co should be interpreted with consideration of potential selection related to retrospective data availability, and these exploratory laboratory associations require validation in larger longitudinal cohorts.

Unlike some earlier studies (13, 26), we did not observe a clear independent association between OHCbl dosage and prognosis. This may reflect the influence of stronger determinants such as age at onset, variation type, and the possibility that even lower doses were sufficient to stabilize the disease. In contrast, longer treatment duration was associated with improved outcomes, supporting the importance of sustained therapy and adherence. Moreover, NBS status, disease onset, HCY levels and the *MMACHC* c.482G>A variant were associated with prognosis, consistent with our previous large-scale analysis of prognostic factors in cblC defect (6). However, given the retrospective observational design, treatment duration may also be influenced by follow-up duration and survivorship. This association therefore warrants further confirmation in prospective longitudinal studies.

Evaluating potential ADRs during long-term OHCbl therapy was another aim of this study. Because these events were retrospectively collected and some manifestations may overlap with the underlying disease or intercurrent conditions, Naranjo scoring was interpreted as a structured but not definitive causality assessment. Subgroup analyses for elevated blood pressure, abdominal pain and diarrhea, and severe allergic reactions, were limited by small sample sizes and should be considered exploratory. Among the reported events, chromaturia was the most common and had the strongest biological plausibility, consistent with the red color of hydroxocobalamin (17). Higher OHCbl dosage was associated with increased odds of overall potential ADRs and chromaturia, supporting a dose-related pattern for this expected event. The *MMACHC* c.482G>A variant was associated with a lower risk of chromaturia, possibly reflecting lower dosage requirements in this genotype (30). Differences in potential ADR frequency across OHCbl brands were also observed. Patients receiving “HYDROXOCOBALAMIN” exhibited a significantly higher risk of chromaturia than those using other brands, likely due to its higher concentration (20–30 mg/ml) and its preference among patients requiring higher dosages. A notably higher incidence of hypertension was observed with “Hycomin”, though this is based on a small number of cases (*n* = 5) and requires further validation in larger cohorts.

This study has several limitations. First, its retrospective design may introduce selection bias related to data availability, recall and assessment variability, residual confounding, and limited causal inference. Standardized developmental or cognitive assessments were not available for all patients, and prognosis was not assessed in a formally blinded manner. Second, several analyses were also constrained by small effective sample sizes, low event counts, incomplete follow-up and limited longitudinal Co measurements. Finally, prospective multicenter studies with standardized assessment of OHCbl exposure, Co kinetics, developmental outcomes, and metabolic monitoring are warranted to validate and extend these findings.

## Conclusion

This study provides one of the largest real-world evaluations of long-term safety profile and prognostic impact of OHCbl therapy. No associations were found between OHCbl dosage, treatment duration, and Co levels with abnormal laboratory findings or poor prognosis. Most potential ADRs, including chromaturia, rash, and psychomotor agitation, were mild, self-limited, and without serious events. Moreover, prolonged treatment was associated with improved metabolic control and favorable outcomes. These findings are consistent with an overall favorable tolerability profile of long-term OHCbl therapy and support sustained disease management with routine monitoring, and future prospective studies will help further clarify the long-term prognostic implications of sustained OHCbl therapy.

## Data Availability

The raw data supporting the conclusions of this article will be made available by the authors, without undue reservation.
