# Supplementary material for: Assessment of blood cobalt levels and safety of long-term hydroxocobalamin therapy in patients with cblC defect
Source: Front Nutr. 2026 Jul 30;13:1843743. doi: 10.3389/fnut.2026.1843743 (PMC13467901; doi:10.3389/fnut.2026.1843743)
Supplement: Supplementary file 1 [file Data_Sheet_1.docx]

Supplementary Material

# 1 Supplementary methods

## 1.1 Detailed clinical and prognostic data collected in this study

Demographics: name, date of birth, sex, height, weight.

Genetic information: MMACHC variants.

Onset of disease: symptom occurrence, onset time, presenting symptoms at onset.

Diagnose information: newborn screening status, phenotype-based diagnosis, family history-triggered.

Treatment profile: duration of treatment, OHCbl dosage, Co concentration.

Metabolic markers: C3, C3/C2, MET, MMA, MCA and HCY at baseline and final follow-up.

Core laboratory panels (Table S1): hematology, urinalysis, liver & cardiac enzymes, renal function, serum protein electrophoresis, thyroid function, heavy metal elements.

Diagnostic imaging studies: liver ultrasound, renal ultrasound, thyroid ultrasound, transthoracic echocardiography, electrocardiography, electroencephalography, magnetic resonance imaging (brain).

Cognitive assessment results (subset of patients): Gesell Developmental Schedules, age-appropriate Wechsler Intelligence Scales: the Wechsler Intelligence Scale for patients ≥ 6 years old and the Wechsler Preschool and Primary Scale of Intelligence for younger participants.

Clinical Functional Assessment: language development assessment, motor function evaluation, academic performance (school-age).

Adverse reaction information: adverse reaction symptoms, dose at onset of adverse reaction, drug trade name, Naranjo scale assessment.

## 1.2 Criteria used for organ-specific involvement and overall prognosis classification

Cardiovascular system: cardiovascular involvement was defined as clinically significant cardiac abnormality documented by transthoracic echocardiography or electrocardiography. When echocardiography and electrocardiography records were unavailable, CK-MB >36 U/L, corresponding to more than three times the institutional upper reference limit, was used as supportive evidence of possible myocardial injury. Isolated CK-MB elevation was interpreted cautiously and was not considered definitive without clinical context.

Hematologic system: hematologic involvement was mainly defined as anemia at the last follow-up. Anemia was determined as hemoglobin below the age-specific lower reference limit used by the clinical laboratory of our hospital. Patients with persistent anemia or anemia requiring clinical follow-up or treatment were classified as having hematologic poor prognosis.

Renal system: renal involvement was defined as clinically significant renal abnormality documented by renal ultrasound or urinalysis at the last follow-up. Abnormal renal ultrasound findings, urinary protein above the institutional laboratory reporting threshold, or urinary erythrocytes or urine hemoglobin above the institutional laboratory reporting threshold were considered supportive evidence of renal involvement. In menstruating female patients, urinary erythrocyte or hemoglobin positivity was interpreted cautiously and was not considered sufficient alone unless confirmed outside the menstrual period or accompanied by proteinuria, renal ultrasound abnormalities, or other clinical evidence.

Hepatic system: hepatic involvement was defined as clinically significant hepatic abnormality documented by liver ultrasound or liver enzyme testing at the last follow-up. Abnormal liver ultrasound findings or AST and/or ALT levels above the institutional laboratory reference range were considered supportive evidence of hepatic involvement. Because aminotransferase levels may be influenced by intercurrent illness or medications, isolated mild elevation was interpreted cautiously in outcome classification.

Nervous system: clinically significant nervous system involvement was defined as developmental, cognitive, motor, neurological, electrophysiological, or neuroimaging abnormality documented by developmental assessment, cognitive testing, neurological examination, electroencephalography, or brain magnetic resonance imaging. Standardized developmental or cognitive assessments, including the Gesell Developmental Schedules and age-appropriate Wechsler Intelligence Scales, were used when available. A Gesell developmental quotient or age-appropriate Wechsler full-scale intelligence quotient <70 was considered evidence of clinically significant developmental or cognitive impairment. For school-age patients without complete standardized testing, persistent language delay, motor dysfunction, poor academic performance, or need for special educational support was considered supportive functional evidence, and was interpreted together with medical records, neurological examination, electroencephalography, or brain magnetic resonance imaging findings.

Endocrine system: endocrine involvement mainly referred to thyroid-related abnormalities in this study. It was defined as clinically significant thyroid abnormality documented by thyroid ultrasound or thyroid function tests at the last follow-up. Thyroid dysfunction was determined by abnormal TSH, FT3, and/or FT4 levels according to the age-specific institutional laboratory reference ranges. Abnormal thyroid ultrasound findings were considered supportive evidence of thyroid-related endocrine involvement. Isolated mild laboratory deviations or nonspecific ultrasound findings without clinical relevance were interpreted cautiously in outcome classification.

Overall prognosis: good prognosis was defined as the absence of documented clinically significant dysfunction in the assessed organ systems and normal developmental status at the last visit. Patients who remained asymptomatic after detection by newborn screening or family-history-based testing were classified as having good prognosis only when no clinically significant organ dysfunction or developmental abnormality was identified from available follow-up data. Poor prognosis was defined as persistent developmental delay, symptom onset with clinically significant sequelae, or dysfunction in one or more organ systems. For patients without complete examinations across all organ systems, overall prognosis was determined by treating clinicians based on available medical records and routine follow-up assessments.

# 2 Supplementary Figures and Tables

## 2.1 Supplementary Figures

**Supplementary Figure 1. Study flowchart.** The study included 379 patients with cobalamin C (cblC) defect. Among them, 270 patients with complete clinical and prognostic data and a subset of 99 patients with cobalt concentration and laboratory data were analyzed for the associations between hydroxocobalamin (OHCbl) treatment, adverse drug reactions (ADRs), prognosis, and laboratory outcomes.

**Supplementary Figure 2. Bar chart showing variables selected through LASSO regression with their corresponding coefficients and the optimal λ value identified by cross-validation.** The selected variables associated with potentially ADRs **(A)** and chromaturia **(B)**. Candidate predictors included OHCbl dosage, treatment duration, sex, age, *MMACHC* variant type, disease onset, NBS status, OHCbl brands and metabolic indicators including C3, C3/C2, MET, MMA, MCA, and HCY. n=126.

**Supplementary Figure 3. LASSO regression for variable selection in predicting potential ADRs.** Coefficient path plots for potentially ADRs **(A)** and chromaturia **(B)** show trajectories of predictor coefficients across -log(λ) values, shrinking toward zero with increasing λ. Cross-validation plots for potentially ADRs **(C)** and chromaturia **(D)** display binomial deviance versus -log(λ), with red dots indicating mean deviance, gray bands representing standard errors, and dashed lines marking optimal λ value. Candidate predictors included OHCbl dosage, treatment duration, sex, age, MMACHC variant type, disease onset, NBS status, OHCbl brands and metabolic indicators including C3, C3/C2, MET, MMA, MCA, and HCY. n=126.

## 2.2 Supplementary Tables

# Supplementary Table 1. Baseline characteristics of the study population

| **Characteristics** | **Cobalt Cohort** | **Clinical Outcome Cohort** | **ADR Cohort** | **p value** |
| --- | --- | --- | --- | --- |
| **Total, n** | **99** | **269** | **379** |  |
| Male sex, n (%) | 56 (56.57) | 149 (55.39) | 209 (55.15) | 0.973 |
| Female sex, n (%) | 43 (43.43) | 120 (44.61) | 170 (44.85) |  |
| Age (months), median (IQR) | 84.00 (29.97-139.20) | 91.93 (53.09-136.7) | 84.00 (47.27-127.4) | 0.3061 |
| MMACHC variants, n | **95** | **233** | **233** |  |
| A | 43 (45.26) | 124 (53.22) | 124 (53.22) | 0.056^*^ |
| B | 17 (17.89) | 41 (17.60) | 41 (17.60) |  |
| C^#^ | 26 (27.37) ^a^ | 35 (15.02) ^b^ | 35 (15.02) |  |
| D | 9 (9.48) | 33 (14.16) | 33 (14.16) |  |
| **Onset of disease, n** | **98** | **263** | **263** |  |
| Without disease onset, n (%) | 29 (29.59) | 60 (22.81) | 60 (22.81) | 0.2164^*^ |
| Disease onset, n (%) | 69 (70.41) | 203 (77.19) | 203 (77.19) |  |
| Early onset, n (%) | 44 (66.67) | 116 (72.96) | 116 (72.96) | 0.4193^*^ |
| Late onset, n (%) | 22 (33.33) | 43 (27.04) | 43 (27.04) |  |
| Onset age (months), median (IQR) | 6.00 (1.37-24.33) | 8.69 (1.44-55.25) | 8.69 (1.44-55.25) | 0.1274^*^ |
| **NBS, n** | **98** | **260** | **260** |  |
| Y, No. (%) | 48 (47.96) | 129 (49.62) | 129 (49.62) | 0.9061^*^ |
| N, No. (%) | 51 (52.04) | 131 (50.38) | 131 (50.38) |  |
| **Treatment** |  |  |  |  |
| Duration of treatment (months), median (IQR) | 42.77 (11.40-90.00) | 76.7 (31.38-114.30) | 76.7 (31.38-114.30) | **0.0003** |
| OHCbl dosage (mg/kg/week), median (IQR) | 1.22 (0.62-1.90) | 1.11 (0.52-1.80) | 1.07 (0.44-1.90) | 0.8889 |
| Cobalt concentration (μg/l), median (IQR) | 21.74 (7.66-38.40) | 21.74 (7.66-38.40) | 21.74 (7.66-38.40) |  |
| **Outcome, n** | **89** | **230** | **230** |  |
| Good prognosis, No. (%) | 25 (28.09) | 74 (32.17) | 74 (32.17) | 0.5032^*^ |
| Poor, No. (%) | 64 (71.91) | 156 (67.83) | 156 (67.83) |  |
| Disorders of the |  |  |  |  |
| Cardiovascular system, No. (%) | 5 (6.33) 74 | 10 (7.09) 131 | 10 (7.09) | >0.9999^*^ |
| Hematologic system, No. (%) | 3 (3.23) 90 | 12 (6.22) 181 | 12 (6.22) | 0.3998^*^ |
| Liver, No. (%) | 5 (5.38) 87 | 12 (7.74) 143 | 12 (7.74) | 0.6075^*^ |
| Kidneys, No. (%) | 5 (5.88) 80 | 7 (4.86) 137 | 7 (4.86) | 0.7648^*^ |
| Endocrine system, No. (%) | 4 (5.88) 64 | 4 (3.17) 122 | 4 (3.17) | 0.4543^*^ |
| Nervous system, No. (%) | 54 (78.26) 15 | 115 (61.17) 73 | 115 (61.17) | **0.0116^*^** |
| **Potential adverse reaction, n** | **83** | **252** | **362** |  |
| None, No. (%)^#^ | 25 (30.12) 58 ^a^ | 46 (18.25) 206 ^ab^ | 63 (17.40) 299 ^b^ | **0.027** |
| Urine red staining, No. (%)^#^ | 57 (67.86) 26 ^a^ | 202 (80.16) 50 ^ab^ | 292 (80.44) 70 ^b^ | **0.046** |
| Psychomotor agitation, No. (%) | 3 (3.61) 80 | 21 (8.33) 231 | 32 (8.84) 330 | 0.239 |
| Localized rash, No. (%) | 3 (3.61) 80 | 14 (5.56) 238 | 20 (5.52) 342 | 0.791 |
| Elevated blood pressure, No. (%) | 0 (0.00) 83 | 3 (1.19) 249 | 5 (1.38) 357 | 0.884 |
| Abdominal pain and diarrhea, No. (%) | 0 (0.00) 83 | 1 (0.40) 251 | 3 (0.83) 359 | 0.788 |
| Severe allergic reaction, No. (%) | 0 (0.00) 83 | 1 (0.40) 251 | 2 (0.55) 360 | >0.9999 |

Abbreviation: n, number; IQR, interquartile range; NBS, newborn screening; OHCbl, hydroxocobalamin.

* The differences between “Cobalt Cohort” and “Clinical Outcome Cohort”.

\# Groups sharing a common superscript letter (a, b) do not differ significantly (p > 0.05), the letter-based comparisons (a,b) are only valid within the same subset row

# Supplementary Table 2. Multivariable linear regression analysis of predictors associated with Co levels (n=75)

| **Predictors** | **B** | **β** | **t** | **p value** | **VIF** | **F** | adj.R^2^ |
| --- | --- | --- | --- | --- | --- | --- | --- |
| Intercept | -1.763 |  | -0.192 | 0.849 |  |  |  |
| OHCbl dosage | 19.966 | 0.696 | 7.033 | **<0.0001** | 1.628 | 10.215 | 0.555 |
| Disease onset | -1.444 | -0.024 | -0.254 | 0.8 | 1.45 |  |  |
| *MMACHC* variants = B | 4.715 | 0.064 | 0.678 | 0.5^a^ | 1.463 |  |  |
| *MMACHC* variants = C | -13.337 | -0.219 | -1.79 | 0.078^a^ | 2.491 |  |  |
| *MMACHC* variants = D | -7.044 | -0.063 | -0.761 | 0.45^a^ | 1.126 |  |  |
| Age | 0.029 | 0.068 | 0.604 | 0.548 | 2.087 |  |  |
| Sex = Female | 7.406 | 0.131 | 1.64 | 0.106^b^ | 1.061 |  |  |
| Duration of treatment | 0.013 | 0.022 | 0.022 | 0.838 | 1.95 |  |  |
| Liver injury | 12.742 | 0.102 | 1.236 | 0.221 | 1.132 |  |  |
| Kidney impairment | 4.798 | 0.034 | 0.373 | 0.71 | 1.342 |  |  |

Abbreviation: Co, cobalt; B, unstandardized regression coefficient; β, standardized regression coefficient; t, t-test statistic; VIF, variance inflation factor; F, F-statistic; adj.R², adjusted R-squared; OHCbl, hydroxocobalamin.

a The differences between “MMACHC variants = B/C/D” and “MMACHC variants = A”.

b The differences between “Sex = Female” and “Sex = male”.

# Supplementary Table 3. Laboratory parameters of cblC patients according to Co levels before and after PSM

|  | **Before PSM** | | | | **After PSM** | | | |
| --- | --- | --- | --- | --- | --- | --- | --- | --- |
| **Parameters** | **Overall** | **Low-Co** | **High-Co** | **p value** | **Overall** | **Low-Co** | **High-Co** | **p value** |
| n | 99 | 46 | 53 |  | 34 | 17 | 17 |  |
| **Hematology** | | | | | | | | |
| WBC (×10⁹/L), median (IQR) | 7.23 (6.19-8.73) | 7.42 (6.39-8.88) | 6.84 (5.74-8.51) | 0.2489 | 7.23 (6.43-8.74) | 7.46 (6.34-8.56) | 6.92 (6.11-8.93) | 0.7572 |
| RBC (×10¹²/L), median (IQR) | 4.48 (4.25-4.76) | 4.69 (4.32-4.94) | 4.36 (4.23-4.64) | **0.0114** | 4.37 (4.20-4.75) | 4.56 (4.16-4.85) | 4.33 (4.24-4.64) | 0.9265 |
| Hb (g/L), median (IQR) | 128.0 (122.3-133.8) | 131.0 (123.0-140.0) | 127.0 (122.0-130.0) | 0.0982 | 128.00 (122.80-136.50) | 130.00 (123.00-140.00) | 126.00 (121.50-131.0) | 0.6859 |
| Hct (%), median (IQR) | 37.50 (36.05-39.70) | 38.50 (35.60-40.98) | 37.20 (36.10-38.65) | 0.0744 | 37.55 (36.35-40.65) | 39.50 (36.40-41.45) | 37.20 (36.05-38.50) | 0.4874 |
| PLT (×10⁹/L), median (IQR) | 312.0 (267.0-382.0) | 313.0 (261.0-387.3) | 309.0 (268.0-377.5) | 0.5491 | 300.50 (264.30-364.30) | 314.00 (260.50-399.00) | 300.0 (263.50-337.00) | 0.5216 |
| MPV (fL), median (IQR) | 7.84 (6.99-8.66) | 8.16 (7.71-9.10) | 7.48 (6.73-8.32) | **0.0055** | 8.18 (7.39-9.13) | 8.34 (8.05-9.34) | 7.44 (6.72-8.50) | 0.0714 |
| NE (×10⁹/L), median (IQR) | 3.09 (2.22-4.08) | 3.12 (1.85-4.26) | 3.00 (2.39-3.84) | 0.5825 | 3.63 (2.75-4.24) | 3.64 (2.74-4.14) | 3.62 (2.80-4.34) | 0.4637 |
| EO (×10⁹/L), median (IQR) | 0.18 (0.10-0.30) | 0.15 (0.093-0.30) | 0.19 (0.11-0.30) | 0.4216 | 0.18 (0.098-0.40) | 0.19 (0.085-0.47) | 0.18 (0.10-0.32) | 0.9099 |
| BA (×10⁹/L), median (IQR) | 0.03 (0.02-0.045) | 0.03 (0.02-0.04) | 0.03 (0.02-0.05) | 0.4416 | 0.03 (0.02-0.04) | 0.03 (0.02-0.04) | 0.03 (0.02-0.045) | 0.2031 |
| MO (×10⁹/L), median (IQR) | 0.51 (0.40-0.61) | 0.52 (0.39-0.62) | 0.50 (0.40-0.61) | 0.8918 | 0.49 (0.41-0.61) | 0.51 (0.39-0.65) | 0.49 (0.41-0.61) | 0.4372 |
| LY (×10⁹/L), median (IQR) | 3.07 (2.62-4.12) | 2.98 (2.59-4.31) | 3.11 (2.55-4.01) | 0.8913 | 2.75 (2.26-3.61) | 2.75 (2.39-4.14) | 2.93 (2.12-3.50) | 0.4037 |
|  |  |  |  |  |  |  |  |  |
| RBC Hb (/μL) | 16 (22.86) | 6 (15.00) | 10 (33.33) | 0.089 | 7 (21.88) 25 | 3 (20.00) 12 | 4 (23.53) 13 | >0.9999 |
| Ketones (mg/dL) | 6 (8.00) | 3 (7.50) | 3 (8.57) | >0.9999 | 3 (9.38) | 1 (6.67) | 2 (11.76) | >0.9999 |
| Protein (mg/dL) | 4 (5.33) | 3 (7.50) | 1 (2.86) | 0.6182 | 2 (6.25) | 1 (6.67) | 1 (5.88) | >0.9999 |
| **Liver & Cardiac Enzymes** | | | | | | | | |
| ALT (U/L), median (IQR) | 14.00 (11.00-20.00) | 15.50 (12.00-22.00) | 14.00 (11.00-18.00) | 0.158 | 12.50 (10.25-15.75) | 13.50 (10.00-19.75) | 12.00 (11.00-14.75) | 0.4901 |
| AST (U/L), median (IQR) | 27.00 (20.00-31.75) | 26.00 (18.00-32.50) | 27.00 (23.00-32.00) | 0.2657 | 24.00 (18.00-27.50) | 24.00 (18.25-27.75) | 24.00 (17.50-27.50) | 0.7147 |
| CK (U/L), median (IQR) | 115.00 (84.50-154.00) | 108.00 (82.00-136.00) | 121.00 (89.50-169.50) | 0.1185 | 102.50 (84.25-122.50) | 103.00 (86.25-129.30) | 100.50 (81.00-117.30) | 0.4459 |
| CK-MB (μg/L), median (IQR) | 15.00 (12.00-19.50) | 14.00 (9.00-18.00) | 15.00 (12.75-20.00) | 0.3786 | 14.00 (11.25-17.75) | 14.00 (9.25-17.75) | 14.00 (12.00-17.75) | 0.8535 |
| LDH (U/L), median (IQR) | 232.00 (199.50-256.50) | 219.00 (189.00-251.00) | 238.50 (210.30-257.50) | **0.0497** | 223.50 (195.50-244.30) | 223.50 (174.80-241.50) | 224.00 (203.30-251.00) | 0.5664 |
| **Renal Function** |  |  |  |  |  |  |  |  |
| BUN (mg/dL), median (IQR) | 4.70 (3.66-5.98) | 4.71 (3.56-5.44) | 4.66 (3.79-6.47) | 0.2068 | 4.79 (3.88-5.98) | 4.79 (3.93-5.32) | 4.76 (3.82-6.47) | 0.3155 |
| Cr (μmol/L), median (IQR) | 31.00 (23.00-40.00) | 32.00 (22.25-41.75) | 31.00 (23.75-37.00) | 0.375 | 33.50 (25.50-40.50) | 33.50 (27.00-41.00) | 32.50 (25.00-37.00) | 0.988 |
| BUN/Cr | 13.25 (9.45-16.37) | 11.26 (8.51-13.64) | 15.08 (11.92-18.23) | **0.0002** | 12.59 (9.78-15.35) | 11.98 (9.31-13.32) | 13.72 (10.02-15.84) | 0.0739 |
| eGFR [ml/(min.1.73 m2)], median (IQR) | 181.30 (170.40-209.70) | 184.80 (175.20-222.80) | 176.50 (162.50-198.10) | 0.0627 | 196.10 (173.70-225.60) | 199.70 (179.10-237.50) | 180.60 (164.80-218.50) | 0.2675 |
| UA (μmol/L), median (IQR) | 247.00 (199.00-321.0) | 260.00 (221.50-378.50 | 215.00 (177.00-276.00) | **0.0027** | 250.50 (202.30-314.80) | 284.50 (221.50-335.00) | 217.00 (180.80-275.50) | **0.0443** |
| **Serum Protein Electrophoresis** | | | | | | | | |
| ALBUMN (g/L), median (IQR) | 63.80 (61.25-66.40) | 63.00 (60.95-66.20) | 64.60 (61.53-67.48) | 0.349 | 62.25 (60.68-64.83) | 62.35 (60.95-64.88) | 62.00 (59.18-64.83) | 0.3815 |
| ALPHA1 (g/L), median (IQR) | 3.40 (3.20-4.00) | 3.40 (3.20-4.30) | 3.40 (3.00-3.90) | 0.134 | 3.40 (3.20-4.25) | 3.40 (3.23-4.30) | 3.50 (3.00-4.00) | 0.533 |
| ALPHA2 (g/L), median (IQR) | 9.18 (6.50-10.15) | 9.30 (7.70-10.20) | 9.08 (6.50-10.05) | 0.7054 | 9.75 (8.93-10.78) | 9.70 (9.03-10.95) | 9.90 (8.30-10.60) | 0.4485 |
| BETA (g/L), median (IQR) | 9.70 (8.85-10.75) | 9.80 (9.10-11.00) | 9.60 (8.65-10.35) | 0.1053 | 10.20 (9.13-11.10) | 10.10 (9.25-11.10) | 10.20 (9.13-11.10) | 0.989 |
| GAMMA (g/L), median (IQR) | 12.50 (9.18-14.65) | 12.45 (9.73-14.50) | 12.50 (8.93-14.88) | 0.9856 | 13.65 (11.70-15.55) | 13.15 (11.70-15.03) | 14.65 (11.48-14.65) | 0.3748 |
| **Thyroid Function** | | | | | | | | |
| TSH (mIU/L), median (IQR) | 2.49 (2.06-3.73) | 2.47 (2.08-4.22) | 2.50 (1.94-3.44) | 0.8074 | 2.33 (2.01-3.10) | 2.10 (1.99-3.97) | 2.60 (2.04-3.10) | >0.9999 |
| FT3 (pmol/L), median (IQR) | 6.53 (5.98-7.00) | 6.54 (5.96-6.79) | 6.51 (5.98-7.13) | 0.568 | 6.25 (5.86-6.59) | 6.07 (5.64-6.54) | 6.49 (6.05-7.11) | 0.4758 |
| FT4 (pmol/L), median (IQR) | 17.53 (16.23-19.20) | 17.31 (16.03-19.16) | 17.64 (16.31-19.25) | 0.7621 | 18.09 (16.97-19.19) | 17.79 (16.75-19.07) | 18.56 (17.65-20.67) | 0.1269 |

Abbreviation: Co, Cobalt; PSM, Propensity score matching; n, number; IQR, interquartile range; WBC, white blood cell count; RBC, red blood cell count; Hb, hemoglobin; Hct, hematocrit; PLT, platelet count; MPV, mean platelet volume; NE, neutrophil count; EO, eosinophil count; BA, basophil count; MO, monocyte count; LY, lymphocyte count; RBC/μL, RBC (per μl); Crystals (Micro.), salt crystals (microscopic); Ketones, urine ketones; Glucose, urine glucose; Protein, urine protein; ALT, alanine aminotransferase; AST, aspartate aminotransferase; CK, creatine kinase; CK-MB, creatine kinase mb isoenzyme; LDH, lactate dehydrogenase; BUN, blood urea nitrogen; Cr, creatinine; UA, uric acid; ALBUMN, albumin; ALPHA1, alpha-1 globulin; ALPHA2, alpha-2 globulin; BETA, beta globulin; GAMMA, gamma globulin; TSH, thyroid-stimulating hormone; FT3, free triiodothyronine; FT4, free thyroxine.

# Supplementary Table 4. Associations of exposure metrics with laboratory incators (BH-FDR-adjusted)

| **Indicators** | **n** | **β_Duration** | **p_Duration** | **q_Duration(BH)** | **β_Dose** | **p_Dose** | **q_Dose(BH)** | **β_Co** | **p_Co** | **q_Co(BH)** |
| --- | --- | --- | --- | --- | --- | --- | --- | --- | --- | --- |
| WBC (×10⁹/L) | 75 | -0.299 | 0.039 | 0.9742 | 0.043 | 0.788 | 0.9840 | 0.045 | 0.799 | 0.9840 |
| RBC (×10¹²/L) | 75 | 0.05 | 0.719 | 0.9840 | -0.099 | 0.524 | 0.9840 | -0.079 | 0.643 | 0.9840 |
| Hb (g/L) | 74 | -0.116 | 0.423 | 0.9840 | 0.01 | 0.948 | 0.9840 | 0.069 | 0.696 | 0.9840 |
| Hct (%) | 75 | -0.013 | 0.924 | 0.9840 | -0.007 | 0.963 | 0.9840 | 0.028 | 0.863 | 0.9840 |
| PLT (×10⁹/L) | 75 | 0.047 | 0.749 | 0.9840 | -0.099 | 0.546 | 0.9840 | 0.154 | 0.393 | 0.9840 |
| MPV (fL) | 73 | -0.259 | 0.088 | 0.9742 | 0.326 | 0.06 | 0.9742 | -0.258 | 0.173 | 0.9742 |
| NE (×10⁹/L) | 75 | -0.214 | 0.129 | 0.9742 | -0.21 | 0.183 | 0.9742 | 0.236 | 0.172 | 0.9742 |
| EO (×10⁹/L) | 75 | 0.048 | 0.765 | 0.9840 | 0.062 | 0.73 | 0.9840 | -0.12 | 0.539 | 0.9840 |
| BA (×10⁹/L) | 75 | 0.274 | 0.1 | 0.9742 | 0.118 | 0.524 | 0.9840 | -0.051 | 0.8 | 0.9840 |
| MO (×10⁹/L) | 75 | -0.119 | 0.48 | 0.9840 | 0.027 | 0.888 | 0.9840 | -0.113 | 0.587 | 0.9840 |
| LY (×10⁹/L) | 73 | -0.146 | 0.221 | 0.9742 | 0.166 | 0.216 | 0.9742 | -0.132 | 0.369 | 0.9840 |
| ALT (U/L) | 74 | 0.066 | 0.69 | 0.9840 | -0.04 | 0.832 | 0.9840 | 0.152 | 0.464 | 0.9840 |
| AST (U/L) | 75 | 0.012 | 0.931 | 0.9840 | -0.219 | 0.171 | 0.9742 | 0.25 | 0.152 | 0.9742 |
| CK (U/L) | 70 | -0.181 | 0.226 | 0.9742 | 0.024 | 0.889 | 0.9840 | 0.074 | 0.693 | 0.9840 |
| CK-MB (μg/L) | 70 | -0.009 | 0.943 | 0.9840 | 0.099 | 0.504 | 0.9840 | -0.129 | 0.422 | 0.9840 |
| LDH (U/L) | 70 | 0.122 | 0.29 | 0.9840 | -0.166 | 0.218 | 0.9742 | 0.011 | 0.938 | 0.9840 |
| BUN (mg/dL) | 73 | 0.194 | 0.122 | 0.9742 | -0.149 | 0.305 | 0.9840 | 0.458 | 0.004 | 0.3480 |
| Cr (μmol/L) | 73 | 0.033 | 0.686 | 0.9840 | -0.049 | 0.606 | 0.9840 | 0.129 | 0.208 | 0.9742 |
| BUN/Cr | 70 | 0.057 | 0.709 | 0.9840 | 0.134 | 0.452 | 0.9840 | 0.201 | 0.298 | 0.9840 |
| eGFR [ml/(min.1.73 m^2^)] | 56 | -0.004 | 0.984 | 0.9840 | 0.118 | 0.586 | 0.9840 | -0.279 | 0.205 | 0.9742 |
| UA (μmol/L) | 74 | -0.193 | 0.128 | 0.9742 | -0.051 | 0.712 | 0.9840 | -0.03 | 0.842 | 0.9840 |
| ALBUMN (g/L) | 73 | -0.223 | 0.067 | 0.9742 | 0.078 | 0.577 | 0.9840 | -0.029 | 0.846 | 0.9840 |
| ALPHA1 (g/L) | 73 | -0.177 | 0.239 | 0.9742 | -0.272 | 0.119 | 0.9742 | -0.029 | 0.877 | 0.9840 |
| ALPHA2 (g/L) | 73 | -0.116 | 0.42 | 0.9840 | -0.113 | 0.497 | 0.9840 | -0.079 | 0.664 | 0.9840 |
| BETA (g/L) | 73 | 0.109 | 0.43 | 0.9840 | -0.052 | 0.746 | 0.9840 | -0.043 | 0.806 | 0.9840 |
| GAMMA (g/L) | 73 | 0.284 | 0.017 | 0.7395 | 0.056 | 0.676 | 0.9840 | 0.063 | 0.668 | 0.9840 |
| TSH (mIU/L) | 54 | -0.28 | 0.145 | 0.9742 | -0.236 | 0.287 | 0.9840 | 0.252 | 0.295 | 0.9840 |
| FT3 (pmol/L) | 50 | -0.176 | 0.391 | 0.9840 | 0.066 | 0.78 | 0.9840 | -0.172 | 0.504 | 0.9840 |
| FT4 (pmol/L) | 51 | -0.154 | 0.425 | 0.9840 | 0.167 | 0.455 | 0.9840 | -0.157 | 0.516 | 0.9840 |

Models were prespecified and adjusted for sex, age, *MMACHC* variant type, disease onset, and metabolic indices (HCY, MMA). BH-FDR adjustment was applied to exposure–indicator tests across the 29 indicators, performed separately for each exposure (Dosage, Co, Duration). p-values for covariates were not adjusted and are not shown.

Abbreviation: see Table S2.

# Supplementary Table 5. Comparison of MMACHC variants and OHCbl brands by type of potential ADRs

|  | **None*** | **Chromaturia*** | **Psychomotor agitation^*^** | **Localized rash^*^** | **Elevated blood pressure^*^** | **Abdominal pain and diarrhea^*^** | **Severe allergic reaction^*^** |
| --- | --- | --- | --- | --- | --- | --- | --- |
| ***MMACHC* variants** | | | | | | | |
| A | 14 (36.84)^a^ | 99 (56.57)^a^ | 13 (76.47)^a^ | 7 (70.00)^a^ | 1 (50.00)^a^ | 1 (100.00)^a^ | 0 (0.00)^a^ |
| B | 4 (10.53)^a^ | 32 (18.29)^a^ | 0 (0.00)^a^ | 2 (20.00)^a^ | 0 (0.00)^a^ | 0 (0.00)^a^ | 0 (0.00)^a^ |
| C | 16 (42.11)^b^ | 17 (9.71)^b^ | 2 (11.76)^a^ | 1 (10.00)^a^ | 0 (0.00)^a^ | 0 (0.00)^a^ | 1 (100.00)^a^ |
| D | 4 (10.53)^a^ | 27(15.43)^a^ | 2 (11.76)^a^ | 0 (0.00)^a^ | 1 (50.00)^a^ | 0 (0.00)^a^ | 0 (0.00)^a^ |
| **OHCbl brands** | | | | | | | |
| Hycomb, Taiwan | 31 (49.21)^a^ | 104 (40.31)^a,b,c^ | 11 (33.33)^a,b^ | 5 (27.78)^a^ | 2 (40.00)^a^ | 2 (66.67)^a^ | 1 (50.00)^a^ |
| Hycomin, Taiwan | 3 (4.76)^a^ | 10 (3.88)^c^ | 5 (15.15)^b,c^ | 1 (5.56)^a^ | 3 (60.00)^b^ | 1 (33.33)^b^ | 1 (50.00)^a^ |
| HYDROXOCOBALAMIN, USA | 26 (41.27)^a^ | 136 (52.71)^b^ | 10 (30.30)^a^ | 10 (55.56)^a^ | 0 (0.00)^a^ | 0 (0.00)^a^ | 0 (0.00)^a^ |
| Other | 3 (4.76)^a^ | 8 (3.10)^b,c^ | 7 (21.21)^c^ | 2 (11.11)^a^ | 0 (0.00)^a^ | 0 (0.00)^a^ | 0 (0.00)^a^ |

Abbreviation: OHCbl, hydroxocobalamin; ADRs, adverse drug reactions;

*Groups sharing a common superscript letter (a, b, c) do not differ significantly (p > 0.05), the letter-based comparisons (a, b, c) are only valid within the same subset column.
